# N-Acetylcysteine for Preventing Acetaminophen-Induced Liver Injury: A Comprehensive Review

**DOI:** 10.3389/fphar.2022.828565

**Published:** 2022-08-10

**Authors:** Anna Licata, Maria Giovanna Minissale, Simona Stankevičiūtė, Judith Sanabria-Cabrera, Maria Isabel Lucena, Raul J Andrade, Piero Luigi Almasio

**Affiliations:** ^1^ Medicina Interna ed Epatologia, Dipartimento di Promozione della Salute, Materno-infantile, di Medicina Interna e Specialistica di Eccellenza “G. D’Alessandro,” PROMISE, Università degli Studi di Palermo, Palermo, Italy; ^2^ Institute of Physiology and Pharmacology, Lithuanian University of Health Sciences, Kaunas, Lithuania; ^3^ Servicio de Farmacología Clínica, Instituto de Investigación Biomédica de Málaga-IBIMA, Hospital Universitario Virgen de la Victoria, Universidad de Málaga, Málaga, Spain; ^4^ UCICEC IBIMA, Plataforma SCReN (Spanish Clinical Research Network), Malaga, Spain; ^5^ Centro de Investigación Biomedica en Red de Enfermedades Hepáticas y Digestivas, CIBERehd, Madrid, Spain; ^6^ Servicio de Aparato Digestivo, Instituto de Investigación Biomédica de Málaga-IBIMA, Hospital Universitario Virgen de la Victoria, Universidad de Málaga, Málaga, Spain

**Keywords:** acetaminophen, drug-induced liver injury, hepatotoxicity, N-acetyl-cysteine, safety

## Abstract

**Aims:** N-Acetylcysteine (NAC) is used as an antidote in acetaminophen (APAP) overdose to prevent and mitigate drug-induced liver injury (DILI). Our objective was to systematically review evidence of the use of NAC as a therapeutic option for APAP overdose and APAP-related DILI in order to define the optimal treatment schedule and timing to start treatment.

**Methods:** Bibliographic databases (PubMed, Web of Science, Embase, and MEDLINE) were searched for retrospective and prospective cohort studies, case series, and clinical trials. The prespecified primary outcomes were DILI-related mortality, hepatotoxicity, and adverse events (AEs).

**Results:** In total, 34 studies of NAC usage in APAP-related DILI cases with 19,580 patients were identified, of which 2,376 patients developed hepatotoxicities. The mortality rate across different studies ranged from 0 to 52%. Large variability of NAC regimens was found, i.e., intravenous (I.V.) (100–150 mg/kg) and oral (70–140 mg/kg), and length of treatment varied—12, 24, or 48 h for I.V. regimen and 72 h for oral administration. The timing of initiation of NAC treatment showed different results in terms of occurrence of hepatotoxicity and mortality; if started within 8 h and no more than 24 h from APAP overdose, either intravenously or orally, NAC administration was efficacious in terms of mortality. The most frequent AEs reported were anaphylactic reactions, followed by cutaneous AEs for the IV route and intestinal AEs for the oral one.

**Conclusion:** NAC improves hepatotoxicity and reduces mortality. Timing of treatment, ranging from 8 to 24 h from APAP overdose, regardless of the regimen or route of administration, is important to prevent or minimize liver damage, particularly in children and in elderly and obese patients.

## Introduction

Drug-induced liver injury (DILI) is a potentially serious hazard of high doses of N-acetyl-para-aminophenol or more simply acetaminophen (APAP), often taken in suicidal attempts, particularly in Anglo-Saxon countries (Bernal et al., 2010). APAP was first introduced in 1955, and the first case of intoxication was reported in 1966. In particular, between 1980 and 1990, there was a very high number of intoxications with APAP, which caused numerous hospitalizations and deaths. Thus, in 1998, the British authorities in the United kingdom were forced to review the packaging. However, as debated by Bateman et al. (2014), after a 16-year evaluation of the effectiveness of pack size restrictions, a very small effect was found. Indeed, APAP intoxications still represent a major public health concern in the United States and Anglo-Saxon countries (Budnitz et al., 2011). In the United States, an intentional overdose of APAP is the most common cause of acute liver failure (ALF) (Ostapowicz et al., 2002); of note, 1 in 20 emergency department visits in 2012 were related to APAP overdose, and approximately 60,000 individuals are hospitalized each year in the United States following intentional or unintentional APAP overdose (Thusius et al., 2019). In the United Kingdom, intentional overdose represents 50% of APAP exposure (Townsend et al., 2001). In a clinical sense, APAP-related DILI shows varying levels of severity, depending on the grade of necrosis of the hepatic central lobular area. APAP is metabolized by the cytochrome P450 2E1 to N-acetyl para-benzoquinone-imine (NAPQI), a metabolite extremely toxic to the liver, which needs sulfhydryl groups to be neutralized. This process depletes glutathione; thus, NAPQI binds to cellular and mitochondrial proteins to form adducts, impairing mitochondrial respiration and generating oxidative stress. When administering N-acetylcysteine (NAC), protection of the liver occurs by increasing the hepatic levels of glutathione, which facilitates the scavenging of reactive metabolites and reactive oxygen species. NAC is a derivative of cysteine, an amino acid able to donate sulfhydryl groups (Akakpo et al., 2020; Heard, 2008; Peterson and Rumack, 1978; Harrison et al., 1991; Mazaleuskaya et al., 2015). Since NAC is able to restore glutathione reserves, it was thought to be used as an antidote to APAP poisoning and overdose since 1974. Several case series of patients who had overdosed from APAP and benefited from NAC treatment began to be published. Different regimens of NAC treatment and routes (intravenous (I.V.) or oral) and timing of NAC administration were extensively evaluated. However, even if oral and I.V. administration schedules are efficient, both can be hampered by severe adverse events (AEs).

Two recently published reports (Chiew et al., 2016; Chiew et al., 2018) highlight the paucity of randomized clinical trials (RCTs) comparing different interventions for APAP overdose and variable routes of administration as well as the low or very low-level quality of evidence that is available. In fact, the current management of APAP overdose involves the administration of NAC, I.V. or orally, which is based mainly on observational studies. Results from a recent meta-analysis (Chiew et al., 2018) indicate that treatment with NAC seems to decrease morbidity and mortality, even if the overall assessment of safety and efficacy of the treatment effect is difficult to evaluate. In particular, limitations of the studies included in the above meta-analysis were their retrospective design, small sample size, lack of information on associated risk factors for hepatotoxicity, under-reporting of timing of ingestion of APAP and NAC, and short follow-up duration.

To overcome some of the abovementioned limitations, observational studies on treatment with NAC following liver damage induced by APAP overdose were reviewed. The aim of this study was to review evidence of use of NAC as a therapeutic option for APAP overdose and APAP-related DILI, defining the optimal treatment schedule as well as the best timing to start treatment, in order to decrease mortality and liver transplantation.

## Material and Methods

The primary source of the studies was PubMed; Web of Science, Embase, and MEDLINE databases were also searched and reviewed without time restriction (January 1970 to December 2019), but it was limited to English language literature. This review was performed following the PRISMA 2020 guidelines.

The medical subject headings used were specific keywords within the title (“acetaminophen,” and/or “N-acetylcysteine,” and/or “poisoning,” and/or “mortality”).

The potentially relevant studies were classified into three subsets and were included if they reported data on the use of NAC as preventing liver damage in patients presenting with APAP overdose and APAP-related DILI and if they reported methodology of study, including design, and outcome measures, such as DILI-related mortality or liver transplantation, hepatotoxicity, and AEs. Subset 1 included retrospective cohort studies, subset 2 included prospective cohort studies, and subset 3 included RCTs comparing different schedules of NAC treatment plus or minus other drugs, with or without an untreated control group.

Studies were considered for inclusion if 1. they enrolled human participants (adults and children), including pregnant women, diagnosed with APAP overdose (meaning, acute ingestion of acetaminophen within 4–24 h of 7.5 g or children with a history of ingesting more than 140 mg/kg); 2. they considered an intervention with NAC I.V. or orally, with no restrictions on dose, timing, and route, with concomitant drugs; 3. primary outcomes were DILI mortality or liver transplantation, hepatotoxicity (defined as ALT >1,000 IU/L) (14), and AEs (graded using the Common Terminology Criteria for Adverse Events) (NCI 2009).

Exclusion criteria were reviews and meta-analyses, animal models, preclinical experimental studies with NAC in APAP-related DILI and acute liver injury due to other etiologies; any pharmacological intervention not including NAC; and studies including specific clinical settings, such as patients with malignancy, HIV, and malnutrition.

### Selection and Eligibility of Studies

After removing duplicates, titles and abstracts were screened independently for eligibility by two reviewers (AL and MM). After reading the titles and abstracts of the identified articles, they acquired the full-text articles of all citations considered to meet the inclusion criteria. These articles were independently examined to verify that they met the inclusion criteria, and disagreements between the two reviewers regarding study eligibility were resolved through discussion with a third author (PA). Further, reference lists of available review articles and primary studies were also checked to identify other studies not found in the literature search.

### Data Collection and Synthesis

The following data were extracted from the included publications: surname of the first author, year of publication, study design, number of included patients, type of NAC intervention, DILI mortality or liver transplantation (N, %), hepatotoxicity (N, %), and AEs (N, %). Relevant results of each study and limitations were also considered. Demographic, clinical, laboratory, and outcome information corresponding to exposure to APAP, resulting in hepatotoxicity, was retrieved from the articles and analyzed.

Findings were summarized in a narrative review. Due to the large heterogeneity of the included studies and limitations reported, a quantitative analysis for only a limited number of variables was possible, such as total number of included patients, rate of DILI mortality, and rate of hepatotoxicity.

## Results

### Eligibility and Exclusion of Studies

**FIGURE 1:**
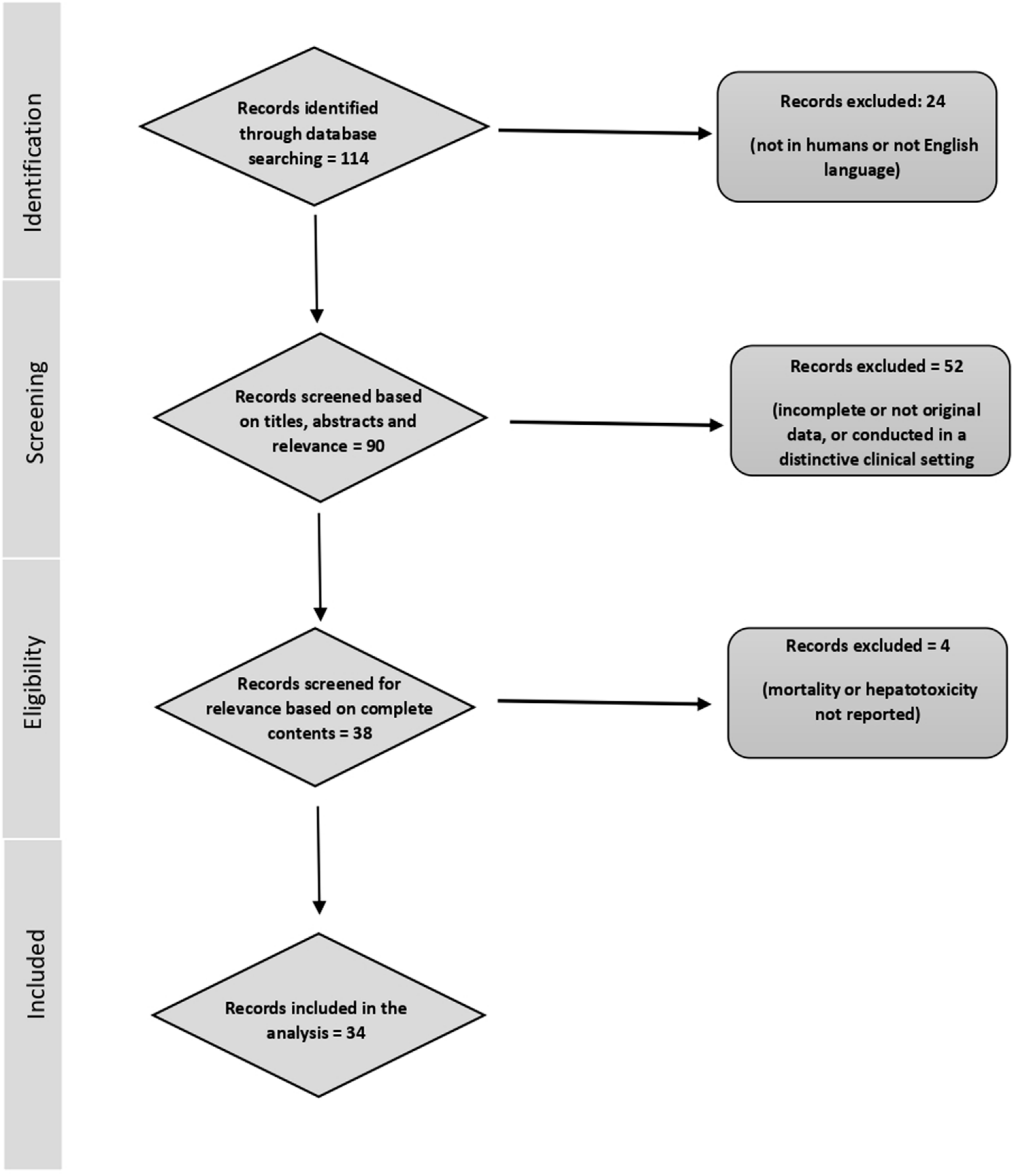
Flow diagram of the report selection of the current analysis.

The applied search strategy led to a total of 114 published studies. Of these, 19 reports were not conducted in the human population and 5 did not meet the language criteria. In the next step, 90 articles were selected because they were of potential interest for the present analysis based on the article titles and abstracts. Of these, 58 were excluded for the following reasons: 31 reported incomplete data or were conducted in a distinctive clinical setting of a patient population, 18 were reviews, and 3 were meta-analyses. In addition, four studies were deemed for potential inclusion, but mortality in cases or controls or hepatotoxicity was not reported (Kerr et al., 2005; Pakravan et al., 2008; Carroll et al., 2015; Isbister et al., 2016). Thus, 34 studies were included in our analysis, and the full text was retrieved and assessed. A flow diagram of the reported selection of studies is shown in Figure 1.

### Study Characteristics

To evaluate the effectiveness of NAC treatment on patients with APAP overdose and APAP-induced liver injury, 34 cohort studies were analyzed: 18 were retrospective, including case series or case reports (Prescott et al., 1979; Harrison et al., 1990; Yip et al., 1998; Buckley et al., 1999; McCormick et al., 2000; Woo et al., 2000, Whyte et al., 2007; Myers et al., 2008; Yarema et al., 2009; Alhelail et al., 2011; Khandelwal et al., 2011; Offerman, 2011; Pettie et al., 2012; Hou et al., 2013; Varney et al., 2014; Curtis and Sivolotti, 2015; Radosevich et al., 2016; Yarema et al., 2018), 13 were prospective (Smilkstein et al., 1988; Rigges et al., 1989; Smilkstein et al., 1991; Perry and Shannon, 1998; Schmidt et al., 2002; Betten et al., 2007; Waring et al., 2008; Doyon and Klein-Schwartz, 2009; Horowitz et al., 1997; Heard et al., 2014; Gheshlaghi et al., 2006; Larson et al., 2005; Rumack, 1984), and 3 were RCTs (Keays et al., 1991; Bateman et al., 2014; Morrison et al., 2019).

A large heterogeneity in the design of the study was found. Among the 18 retrospective studies, 1 was multicentric (Varney et al., 2014) and 5 were case series (Buckley et al., 1999; Pettie et al., 2012; Hou et al., 2013; Curtis and Sivilotti, 2015) or reviews of medical records not reporting information on AEs. Retrospective cohort studies and case–control studies were affected by different selection biases, i.e., difficulties in determining the APAP level in enrolled patients, small sample size, long course of data collection, short follow-up duration, lack of external validation, presence of confounding factors, presence of historical controls, long interval between data collection and publication, and lack of comments on the clinical course of untreated patients (Mc Cormick et al., 2000; Whyte et al., 2007; Offerman, 2011; Hou et al., 2013; Myers et al., 2008; Yarema et al., 2009; Alhelail et al., 2011; Radosevich et al., 2016; Yarema et al., 2018).

Among the 13 prospective studies, 2 were single-arm open-label trials (Smilkestein et al., 1991; Heard et al., 2014) without randomization or a comparator group. Among the prospective cohort studies, two studies on a pediatric population were included; the first study by Rumack (Rumack, 1984) included 417 children aged 5 years or younger, and the second study by Perry and Shannon (Perry and Shannon, 1998), in 1998, included 54 adolescent patients (older than 12 years), in which recruitment was carried out for 10 years and historical controls were considered. Further, in this subset, two studies on pregnant women were also included (Rigges et al., 1989; Horowitz et al., 1997).

Only three RCTs were included in our analysis (Keays et al., 1991; Bateman et al., 2014; Morrison et al., 2019), and all were randomized with a control group. In these three studies, concomitant drugs plus NAC administration were considered either to prevent AEs (Bateman et al., 2014) or to increase the antioxidant effect of NAC itself (Morrison et al., 2019).

Regarding the administration of NAC, either I.V. or orally, a large variability was found among included studies. NAC regimens ranged from 100 to 150 mg/kg (I.V. administration) and from 70 to 140 mg/kg (oral administration). Different schedules and durations of treatment—12, 24, and 48 h for I.V. and 72 h for oral administration—were found. In addition, timing of NAC initiation, within 8 h and no more than 24 h after APAP overdose, either intravenously or orally, showed different results in terms of hepatotoxicity occurrence and mortality.

Alcohol intake or pre-existing liver disease were noted in two studies. In a prospective study (Schmidt et al., 2002), chronic alcohol abuse was an independent risk factor that could be counteracted by concomitant acute alcohol ingestion. In a retrospective study (Myers et al., 2008) in which 1,543 patients were hospitalized for APAP overdose, 34% were alcohol abusers and 3% had liver disease. It is noteworthy that some studies, either retrospective or prospective, have been carried out in specific clinical settings of patients, such as pregnant women (Betten et al., 2007; Hou et al., 2013), children (Rumack 1984; Perry and Shannon, 1998), or obese patients (Weighting >100 kg) (Varney et al., 2014; Radosevich et al., 2016).

The outcomes evaluated were DILI mortality or liver transplantation, hepatotoxicity due to APAP, and finally, AEs. In the majority of studies, this information was not systematically recorded. In fact, there was a large heterogeneity in the data reported, especially among the retrospective studies, in which information regarding AEs was reported only in six studies. Efficacy (prevention of mortality or liver transplantation and prevention or management of hepatotoxicity) was reported in all included studies, while safety (presence of any AEs) was reported in nearly half of the studies (16 out of 34) (Keays et al., 1991; Smilkstein et al., 1991; Perry and Shannon, 1998; Yip et al., 1998; Buckley et al., 1999; Gheshlaghi, 2006; Betten et al., 2007; Whyte et al., 2007; Waring et al., 2008; Yarema et al., 2009; Bateman et al., 2014; Heard et al., 2014; Varney et al., 2014; Radosevich et al., 2016; Yarema et al., 2018; Morrison et al., 2019). The design and characteristics of all included studies are mentioned in Tables 1–3. Heterogeneity and quality assessment of the included studies are reported in Table 4, Table 5, and Table 6).

**TABLE 1:** Study design, characteristics, and outcomes of 18 retrospective studies included in the analysis.

Author (year)Country	Study design/patient number Age/sex (male %)	Intervention	Mortality, OLT (N, %)	Hepatotoxicity (N/Tot, %)	AEs (N, %)
Prescott et al. (1979)	Retrospective study involving 100 patients	NAC I.V.	2/100 (2%)	21/100 (21%)	none
United Kingdom	13–82 years	150 mg/kg→ 50 mg/kg > 100 mg/kg less than 10 h *vs*. more than 10 h			
	Male 33%				
Harrison et al. (1990)	Retrospective study involving 43 patients	NAC I.V. standard dose	15/43 (34.5%)	28/43 (65%)	none
United Kingdom	Adult (>18 years)				
	Male 35%				
Yip et al. (1998)	Retrospective consecutive case series of 1131 patients	NAC oral and I.V. NAC I.V. administration of the oral preparation (76)	3/1131 (0.26%)	0	4/76 AEs (5.3%)
United States	Newborn—67 years				(erythema, itching)
	Male 33%				
Buckley et al. (1999)	Case series, 205 patients	NAC I.V.	2/205 (0.97%)	30/205 (14%)	12 AEs (6%)
Australia	0–89 years	300 mg/kg for 20 h			Flushing and urticaria. Anaphylactic reactions
	Male 36%				
McCormick et al. (2000)	Not reported, 110 patients	NAC oral	1/110 (0.90%)	4/110 (3.6%)	NR
Ireland	13–85 years				
	Male 44%				
Woo et al. (2000)	Retrospective cohort study	NAC oral <21 h; 20–21 h; >21 h	0	6/75 (8%)	NR
California, United States	75 patients				
	12–76 years				
	Male 34%				
Whyte et al. (2007)	Retrospective analysis	NAC I.V. <8 h *vs*. >8 h	12/399 (3%)	53/399 (13%)	37 (9, 3%) AEs
Australia	399 patients				
	0–96 years				(anaphylactic, nausea, vomiting)
	Male 35%				
Myers et al. (2008)	Retrospective, 290 patients	NR	15/290 (5%)	70/290 (24%)	NR
Canada	0–96 years				
	Male 32%				
Yarema et al. (2009)	Retrospective with historical controls, 4048 patients (2086 I.V. 1962 os)	NAC oral *vs.* I.V.	11 (3 + 8)/4048	599/4084 (14%): 89/2086 IV (13.8%)	147/2086 (7%) anaphylactic reactions
Canada	Adult >18 years		(0.27%)	310/1962 OS (15.8%)	
Alhelail et al. (2011)	Retrospective, 119 patients	NAC oral and I.V.	6/119 (5%)	44/119 (36%)	NR
Saudi Arabia	Adult >18 years				
	Male 36%				
Khandelwal et al. (2011)	Retrospective, 210 patients	NR	103/210 (49%)	187/210 (89%)	NR
United States	Adult >18 years				
	Male <50%				
Offerman (2011)	Retrospective cohort, 428 patients	Oral *vs.* I.V. NAC	4/428 (0.9%)	4/428 (0.9%)	NR
United States	Adult >18 years				
	Male 27%				
Pettie et al. (2012)	Retrospective, case note review, 71 patients	NAC I.V.	0	NR	NR
United Kingdom	14–94 years				
	Male 30%				
Hou et al. (2013)	Medical records, 147 patients	Route of administration of NAC not reported	1/147 (0.68%)	15/147 (10%)	NR
Taiwan	Adult >18 years				
	Male 18%				
Varney et al. (2014)	Multileft retrospective, 37 patients	NAC 140 mg/kg oral	4/37 (10%)	12/37 (32%)	8 (21%) non-serious AEs, (nausea, vomiting)
United States	Adult >18 years				
	Male 46%	150 mg/kg I.V.			
Curtis and Sivilotti, (2015)	Observational case series, 68 patients	NAC I.V.	9/68 (13%)	28/68 (41%)	NR
Canada	Adult >18 years				
	Male 37%				
Radosevich et al. (2016)	Retrospective cohort, 80 patients	NAC I.V.	5/80 (6,25%)	26/80 (32%)	0 (0%)
Arizona, United States	Adult >18 years	150 mg/kg→ 50 mg/kg > 100 mg/kg			
	Male 30%				
Yarema et al. (2018)	Retrospective study, 6450 patients	NAC I.V. <21 h; 20–21 h; >21 h	136/6450 (2.1%)	NR	528 (8,2%) anaphylactic reactions, mainly cutaneous
Canada	Adult >18 years				
	Male 30%				

**TABLE 2:** Study design characteristics and outcomes of 13 prospective studies included in the analysis.

Author (year)Country	Study design/patient numberAge Sex (male %)	Intervention	Mortality, OLT (N, %)	Hepatotoxicity (N/Tot)	AEs (N, %)
Smilkstein et al. (1988)	Multicenter study involving 2540 patients	NAC oral	28/2540 (1, 1%)	611/2540 (24%)	NR
Colorado, United States	10–30 years				
	Male 30%	140 → 70 mg/kg 10–24 h			
Rigges et al. (1989)	Prospective study of 60 patients	NAC oral	1/60 (1.6%)	24/60 (49%)	NR
Colorado, United States	Adults (>18 years)				
	Female 100% pregnant	140 mg/kg→ 70 mg/kg 72 h			
Smilkstein et al. (1991)	Non-RCT of 179 patients	NAC I.V.	2/179 (1.11%)	39/179 (21%)	32 (17%) AEs (erythema, urticaria)
Colorado, United States	Adult (>18 years)				
	Children <5–10 years	48 h:140 mg/kg→ 70 mg/kg			
	Male 33%				
Perry and Shannon, (1998)	Prospective with historical controls	NAC I.V. *vs*. oral	0	6/54 (11%)	2 (7%) NAC I.V.
Massachusetts, United States	54 patients	25 with NAC I.V.			2 (6%) oral
	Pediatric (<18 years)	29 with NAC oral (controls)			(rash, urticaria, fever, anaphylaxis)
	Female 90%				28 (vomiting)
Schmidt et al. (2002)	Prospective study of 645 patients	NAC I.V.	51/645 (7.9%)	28/645 (4.3%)	NR
Denmark	12–86 years				
	Male 44%	150 mg/kg→ 50 mg/kg > 100 mg/kg (<12, 24, 48 h)			
Betten (2007)	Prospective observational, 205 patients	NAC oral <48 h	0	0	8 (4%) AEs, abdominal pain and vomiting
United States	1–81 years				
	Male 33%				
Waring et al. (2008)	Prospective, 362 patients	NAC I.V.	0	194/362 (53%)	147 (40, 6%) with 54 (14.9%) anaphylactic reactions
	Adults (>18 years, mean age 35 years)				
United Kingdom	Male 34%	150 mg/kg→ 50 mg/kg > 100 mg/kg			
Doyon and Klein-Schwartz (2009)	Prospective, cohort, 77 patients	NAC I.V.	2/77 (2.5%)	4/77 (5%)	NR
	13–75 years				
	Male 33%				
United States					
Horowitz et al. (1997)	Case series involving 4 patients	NAC oral and I.V.	0	2/4 (50%)	NR
Colorado, United States	Adult (>18 years)				
	Female 100% pregnant				
Heard et al. (2014)	Multicenter, single-arm, open-label clinical trial of 309 young patients and children (mean age 21 years)	NAC I.V.	1/309 (0.3%)	(56/309) 18%	28, 8%
United States	Male 33%	140 mg/kg→ 70 mg/kg		Overall	Nausea, vomiting, flushing
		Every 4 h for 12 doses		3.4% patients treated within 10 h	
Gheshlaghi (2006)	Case series and descriptive analytic study of 173 patients	NAC I.V.	0	-	44, 5% anaphylactoid reaction, 63% nausea and vomiting, 30% flashing, 26% bronchospasm, 23% vertigo, 32% skin rash
Iran	15–30 years				
	Male 40%	150 mg/kg→ 50 mg/kg→100 mg/kg			
Larson et al. (2005)	Prospective study	NAC	74 (26%) died without OLT	250 (91%)	NR
Pennsylvania, United States	Involving 275 patients	In 231 patients	18 (6%) lived with OLT		
	Adult (>18 years)		5 (2%) died with OLT		
	Male 36%				
Rumack (1984)	Multicenter, open, prospective study of 417 patients	NAC os	0	3/417 (0, 71%)	NR
Colorado, United States	Pediatric	140 mg/kg--> 70 mg/kg			
	Less than 5 years	Every 4 h for 17 doses			

**TABLE 3:** Study design characteristics and outcomes of three clinical trials included in the analysis.

Author (Year)Country	Study design/patient number Age Sex (male %)	Intervention	Mortality, OLT (N, %)	Hepatotoxicity (N/Tot)	AEs (N, %)
Keays et al. (1991)	Prospective RCT involving 50 patients: 25 NAC vs. 25 control	NAC I.V. 150 mg/kg →50 mg/kg + intensive care	13/25 NAC (52%)	-	0
London	Adult (>18 years)	Control group dextrose 5% + intensive care	20/25 control group (80%)		
	Male 48%				
Bateman et al. (2014)	Double-blind, randomized study involving 222 patients	NAC I.V.	0	13/101	Vomiting: 71/109
United Kingdom	110 standard	Standard: 150 mg/kg→ 50 mg/kg→100 mg/kg (20–25 h)		Standard vs.	Standard vs. 39/108 shorter
	112 shorter	Shorter: 100 mg/kg → 200 mg/kg (12 h)		9/100 shorter	45/109 ondansetron*Vs*. 65/108 placebo
	Adult (>18 years)	With or without I.V. ondansetron pre-treatment or placebo		16/100 ondansetron	
	Male 40%			*vs.* 6/100 placebo	Anaphylactoid reactions: 31 standard *vs*. 5 shorter
	
Morrison et al. (2019)	Randomized study involving 24 patients	6: NAC alone	0	2/6 (NAC alone)	Serious adverse events
United Kingdom	Adult (>18 years)	6: NAC I.V. + calmangafodipir 2 mmol/kg		*vs*.	2/6 (NAC alone)
	Male 45%	6: NAC + calmangafodipir 5 mmol/kg		0/18 (NAC + calmangafodipir)	vs.
		6: NAC + calmangafodipir 10 mmol/kg			4/6 (NAC +calmangafodipir 2) vs.
					2/6 (NAC + calmangafodipir 5) vs.
					3/6 (NAC + calmangafodipir 10)

NAC, N-acetylcysteine; AE, adverse events; LT, liver transplantation; I.V., intravenous; DILI, drug-induced liver injury; NR, not reported.

**TABLE 4:** | Heterogeneity and quality assessment of 18 retrospective studies included in the analysis.

Author (Year)	Study design/patient number	Results	Comments/limitations
Prescott et al. (1979)	Retrospective study involving 100 patients	Efficacy of NAC diminished progressively and treatment after 15 h was completely ineffective	This was one of the first studies on NAC for paracetamol poisoning, and this study described the results of only 100 cases
		Compared to cysteine and methionine, NAC was more effective and safer	
Harrison et al. (1990)	Retrospective study involving 43 patients	This study considered fulminant hepatitis and good outcome after late administration of NAC	Results needed to be confirmed in a prospective, randomized, placebo-controlled trial
Yip et al. (1998)	Retrospective consecutive case series involving 1131 patients	I.V. administration of the oral NAC preparation appears to have limited adverse effects and offers another mechanism of delivery of the potentially lifesaving NAC when oral administration is not possible	Retrospective nature of the study
			Human error during processing data
Buckley et al. (1999)	Case series involving 205 patients	A shorter hospital stay, patient and doctor convenience, and the concerns over the reduction in the bioavailability of oral NAC by charcoal and vomiting make I.V. NAC preferable for most patients with acetaminophen poisoning	
McCormick et al. (2000)	Not reported involving 110 patients	Delays in treatment of APAP overdose are common and may be clinically important in the small minority of patients with significant liver injury; it is possible that oral administration of NAC (that can be given immediately) may reduce these delays	The design of the study did not allow the determination of the cause of the delays in the administration of NAC
			Only one of the two hospitals chosen had APAP level testing
Woo et al. (2000)	Retrospective cohort study involving 75 patients	Patients without evidence of hepatotoxicity within 36 h of an acute overdose can be safely and effectively treated with a shorter course of oral NAC therapy (24 h or less)	Retrospective study
			Small number of patients
Whyte et al. (2007)	Retrospective analysis involving 399 patients	Hepatotoxicity was significantly less likely when I.V. NAC was administered within 8 h after APAP ingestion compared with longer intervals	This study could not exclude certain sources of biases, including temporal changes over the 16-year course of data collection in the use of I.V. NAC and low ascertainment of mild, self-limiting reactions to I.V. NAC
Myers et al. (2008)	Retrospective, 290 patients	APAP overdose had a relatively benign short-term course but was associated with substantial long-term mortality caused by preventable conditions, such as unintentional overdoses, alcohol abuse, and underlying liver disease	Low accuracy of administrative data for APAP overdose, overdose intent, liver disease, and alcohol abuse
			Lack of external validation
			Data only in hospitalized patients
Yarema et al. (2009)	Retrospective with historical controls	The risk of hepatotoxicity differed between the 20-h and 72-h protocols according to the time to initiation of acetylcysteine. It favored the 20-h protocol for patients presenting early and favored the 72-h protocol for patients presenting late after acute acetaminophen overdose	Comparison of retrospective vs. prospective
	4048 patients (2086 I.V.–1962 os)		Confounding: pre-existing liver disease
Alhelail et al. (2011)	Retrospective, 119 patients	Patients with repeated supratherapeutic ingestion of APAP who developed hepatotoxicity presented with abnormal ALT levels. A history of alcoholism and an elevated creatinine level at presentation are markers of increased risk for hepatotoxicity and death	Retrospective study
			Considered only variable included in the original medical record
			Lack of comments on course of untreated patients
Khandelwal et al. (2011)	Retrospective, 210 patients	This study confirms and extends previous observations regarding the high (18%) prevalence of unrecognized or uncertain acetaminophen toxicity among subjects with indeterminate acute liver failure. NAC use was limited presumably because of the lack of a specific diagnosis of APAP toxicity.	Retrospective reports
Offerman (2011)	Retrospective cohort, 428 patients	Use of I.V. NAC did not impact hospital length of stay	Data on the exact time of ingestion and the reported dose of APAP ingested were not collected
Pettie et al. (2012)	Retrospective, case note review, 71 patients	Implementation of an integrated care pathway (ICP) for APAP poisoning significantly improved patient management and helped to standardize interprofessional decision-making in this challenging patient group, improving patient outcomes	Results of the study are based on the introduction of the ICP within a specialist unit.
			Retrospective reports
			Cohorts were unequal in size
Hou et al. (2013)	Medical records, 147 patients	The analytical data revealed that toxic hepatitis was common after APAP overdose, but the study failed to identify any significant risk factors for complications after ingestion. The favorable outcomes depend probably on a prompt diagnosis of poisoning and the immediate institution of detoxification protocols.	Small sample size
			Short follow-up duration
			Retrospective
Varney et al. (2014)	Multileft, retrospective, 37 patients	This study considered obese patients (>100 kg). Clinicians used a weight-based NAC dose rather than a maximum weight cut-off dose. Hepatotoxicity was common in our cohort. AEs were relatively common but not serious	Small number of heavy patients
			Patients were not massively obese
			Lack of height and BMI
			Observational nature of the study
Curtis and Sivilotti (2015)	Observational case series, 68 patients	This study described AST and ALT rise and fall following acetaminophen overdose and discontinuation of NAC	Retrospective nature of the study
			Limited number of deaths precluded a detailed comparison of AST/ALT in fatal events
Radosevich et al. (2016)	Retrospective cohort, 80 patients	Obese and nonobese patients being treated with I.V. NAC for APAP toxicity experienced similar rates of hepatotoxicity	Retrospective study
			Limited data regarding APAP ingestion history
			Limited number of obese patients
Yarema et al. (2018)	Retrospective cohort study, 6450 patients	Anaphylactic reactions to the 21-h I.V. NAC protocol were uncommon and involved primarily cutaneous symptoms. Being female and having taken a single, acute overdose was associated with more severe reactions, whereas higher serum APAP concentrations were associated with fewer reactions.	Retrospective study, with no available data for analysis (data about risk factors, such as asthma, atopy, family history of allergy, previous reactions)
		They were not strong enough to impact the current clinical decision-making surrounding the initiation of NAC	Long interval between data collection and publication
			Hospitalized patients, not from the emergency department

**TABLE 5:** | Heterogeneity and quality assessment of 13 prospective studies included in the analysis.

Author (year)	Study design/patient number	Results	Comments/limitations
Smilkstein et al. (1988)	Multileft involving 2540	The 72-h regimen of oral NAC was as effective as the 20-h I.V. regimen and may be superior when treatment was delayed	There may be a selection bias in the choice of patient-associated factors, which may increase or diminish the potential for hepatotoxicity (e.g., ethanol or other drugs were not analyzed)
	patients		
Rigges et al. (1989)	Prospective cohort study involving 60 patients	There was a statistically significant correlation between the time to a loading dose of NAC and pregnancy outcomes, with an increase in the incidence of spontaneous abortion or fetal death when treatment began late. Pregnant women who take an acetaminophen overdose and have a potentially toxic serum level should be treated with NAC as early as possible	This is the only study involving pregnant women
Smilkstein et al. (1991)	Nonrandomized trial involving 179 patients	48-h I.V. NAC protocol is equal to 72-h oral and 20-h I.V. treatment protocols when started early and superior to the 20-h I.V. regimen when treatment is delayed. NAC-induced adverse effects were dose-related	There was no randomization of treatment
			Analysis of other factors theorized to affect acetaminophen toxicity was limited (ethanol use, other drugs, nutritional status)
Perry and Shannon (1998)	Prospective with historical controls of 54 patients	This study considered pediatric patients	
		Route of administration was discretionary	
Schmidt et al. (2002)	Prospective study involving 645 patients	Time to NAC was the major risk factor in acetaminophen-induced hepatotoxicity and mortality. Chronic alcohol abuse was an independent risk factor, which could be counteracted by concomitant acute alcohol ingestion	Partly retrospective design
Betten et al. (2007)	Prospective observational, 205 patients	A shortened duration of NAC treatment (20–48 h) may be an effective option in individuals considered to be at no further risk of developing liver toxicity according to laboratory criteria (serum APAP undetectable and liver test results normal) before NAC discontinuation	Limited sample size of patients
			Some patients were unable to be contacted after discharge
Waring et al. (2008)	Prospective, 362 patients	High serum acetaminophen concentrations were associated with fewer anaphylactic reactions, suggesting that these might in some way be protective	Study design did not address the biological bases for an association between acetaminophen concentrations and anaphylactic reactions
Doyon and Klein-Schwartz (2009)	Prospective, cohort, 77 patients	Hepatotoxicity developed in 5.2% of cases, suggesting that the 21-h I.V. NAC regimen is suboptimal in some patients. In addition to high initial plasma APAP concentrations, APAP product formulation and persistently elevated plasma APAP concentrations were identified as factors possibly associated with developing hepatotoxicity	Retrospective study
			Small number of patients
			Reporting to the poisoning was voluntary, with possible bias
			Reviewers were not blinded
Horowitz et al. (1997)	Case series involving 4 patients	This study considered pregnant women and their infants discharged without liver injury	Pregnant and placental transfer of NAC
		NAC was detected in the blood of infants and there was no evidence of APAP-related toxicity	
Heard et al. (2014)	Multileft, single-arm, open-label clinical trial, involving 309 patients	APAP-overdosed patients treated with I.V. acetylcysteine administered as a 140 mg/kg loading dose, followed by 70 mg/kg every 4 h for 12 doses, had a low rate of hepatotoxicity and few adverse events	The study design did not have a comparator group. There is a long interval between data collection and publication
Gheshlaghi (2006)	Case series anterograde and descriptive analytic study, involving 173 patients	Different atopic diseases must be considered a risk factor in the development of side effects to I.V. NAC therapy	It is not a case-controlled study
Larson et al. (2005)	Prospective study, involving 275 patients	Detailed prospective data were gathered on 662 consecutive patients fulfilling standard criteria for ALF (coagulopathy and encephalopathy), from which 275 (42%) were determined to result from APAP liver injury. Unintentional overdoses accounted for 131 (48%) cases, intentional 122 (44%), and 22 (8%) were of unknown intent. Transplant-free survival rate and rate of liver transplantation were similar between intentional and unintentional groups	APAP hepatotoxicity far exceeds other causes of acute liver failure in the United States. Susceptible patients have concomitant depression, chronic pain, and alcohol or narcotic use, and/or take several preparations simultaneously
Rumack (1984)	Multileft, open, prospective study, involving 417 children	Even though APAP has been frequently ingested, it infrequently has serious consequences. Alcohol seems to have some degree of hepatoprotection when ingested simultaneously. Miscellaneous additional ingestants increase the risk of lethargy and result in a higher transient elevation of AST level	The purpose of this study was to evaluate the nature of toxic reactions to a substantial overdose of acetaminophen in children aged 5 years or younger

**TABLE 6:** Heterogeneity and quality assessment of three clinical trials included in the analysis.

Author (year)	Study design/patient number	Results	Comments/limitations
Keays et al. (1991)	Prospective randomized controlled study, involving 50 patients: 25 NAC–25 control	NAC is safe and effective in fulminant hepatic failure after APAP overdose	
Bateman (2014)	Double-blind randomized study, involving 222 patients	In patients with APAP poisoning, a 12-h modified NAC regimen resulted in less vomiting, fewer anaphylactoid reactions, and reduced need for treatment interruption	The open nature of the comparison might have led to observer bias in the assessment of adverse reactions.
	110 standard		The trial was not sufficiently powered to show noninferiority of the modified acetylcysteine regimen for the prevention of hepatotoxic effects
	112 shorter		
Morrison et al. (2019)	Randomized study, involving 24 patients	Calmangafodipir was tolerated when combined with NAC and may reduce biomarkers of paracetamol toxicity	The patients were not stratified at randomization by the risk of developing a liver injury. There was a small patient number

NAC, N-acetylcysteine; AE, adverse events; LT, liver transplantation; I.V., intravenous; DILI, drug-induced liver injury; NR, not reported.

### Clinical Features, Severity, and Outcomes According to the Route of N-Acetylcysteine Administration

In cases of APAP overdose, various factors determine the effectiveness of NAC treatment. The time elapsed between starting treatment and APAP overdose is certainly the most relevant (Rumack et al., 1981). However, other factors, such as chronic alcohol ingestion, underlying liver disease, and impaired renal function should be considered in determining the patient’s favorable or unfavorable outcome (Alhelail et al., 2011). In addition, other predisposing conditions, such as being female, having asthma or atopy, and having a history of allergy, could severely impact the patient’s clinical outcome (Yarema et al., 2018) due to the difficulties linked to I.V. NAC treatment, which is rapid and certainly the most useful in cases of larger overdoses (Herad et al., 2014), due to delayed arrival of the patient at the emergency ward (Smilkestein et al., 1988; Smilkestein et al., 1991; Buckley et al., 1999), or due to fulminant hepatitis (Harrison et al., 1990).

In the retrieved studies, NAC was administrated both intravenously and orally with different regimens and without restrictions on dose, timing, and route of administration. The most frequent schedule of NAC, if administered intravenously, reported in 18 studies (Prescott et al., 1979; Harrison et al., 1990; Keays et al., 1991; Smilkstein et al., 1991; Buckley et al., 1999; Schmidt et al., 2002; Gheshlaghi, 2006; Whyte et al., 2007; Waring et al., 2008; Doyon and Klein-Scwartz, 2009; Pettie et al., 2012; Bateman et al., 2014; Heard et al., 2014; Curtis and Sivilotti, 2015; Radosevich et al., 2016; Yarema et al., 2018; Morrison et al., 2019), was 150 mg/kg in 200 ml of 5% dextrose as a loading dose in a 15 to 60-min (1 h) infusion, followed by 50 mg/kg in 500 ml of 5% dextrose for 4 h, and finally 100 mg/kg in 1 L of 5% dextrose over the next 16 h. In the study by Heard et al. (Heard et al., 2014), APAP-overdosed patients treated with NAC administered intravenously as a 140 mg/kg loading dose, followed by 70 mg/kg every 4 h for 12 doses, had a low rate of hepatotoxicity and few AEs (18% hepatotoxicity, 29% AEs). This protocol delivers a higher dose of NAC, which may be useful in selected cases involving very large APAP overdoses. In fact, this protocol has been used in the study by Radosevich et al., in which obese patients with a weight of >100 kg were included (Radosevich et al., 2016). Both studies reporting higher doses of administered NAC showed a higher incidence of not serious AEs, 21% and 28.9%, respectively.

An oral NAC regimen was reported in five studies (Smilkstein et al., 1988; Rigges et al., 1989; McCormick et al., 2000; Woo et al., 2000; Betten et al., 2007), and it was administered at 140 mg/kg as a loading dose, followed by 17 maintenance doses of 70 mg/kg every 4 h (total treatment time of 72 h). One study reported a schedule of oral NAC administered for less than 24 h in patients without hepatotoxicity within 36 h from APAP overdose (Woo et al., 2000). In the study by McCormick et al. (McCormick et al., 2000), involving 110 patients, they suggested that oral treatment, which can be administered immediately, allows overcoming unnecessary delays in treating patients who have been subjected to APAP overdose. Betten et al. affirm that a shortened duration of treatment with oral NAC (<48 h) may be an effective treatment option in individuals considered to be at no further risk of developing liver toxicity according to the fulfillment of appropriate laboratory criteria (APAP undetectable and normal liver function tests) before NAC discontinuation (Betten et al., 2007). Thus, in the study by Yarema et al., the risk of hepatotoxicity differed between the 20-h and 72-h protocols according to the time to initiation of NAC. It favored the 20-h protocol for patients presenting early and the 72-h protocol for patients presenting late after acute acetaminophen overdose.

In six studies (Yarema et al., 2009; Alhelail et al., 2011; Offerman, 2011; Varney et al., 2014; Perry and Shannon, 1998; Horowitz et al., 1997), there was a comparison between two routes of NAC administration (I.V. and oral), and in Yip’s study (Yip et al., 1998) of 76 patients, the oral preparation was administered as an I.V. infusion. In two studies, the schedule of treatment was not reported (Myers et al., 2008; Hou et al., 2013).

I.V. administration directly introduces NAC into the portal circulation, allowing faster availability of sulfhydryl groups, after the hepatic passage. Although I.V. administration of NAC within 8–10 h of APAP ingestion reduces the risk of hepatotoxicity by 58–1.6% (Prescott et al., 1979), patients will still benefit from NAC treatment regardless of the route of administration, especially if treated early. In this regard, Smilkstein compared a 48-h I.V. administration (980 mg/kg) with a 21-h I.V. administration (300 mg/kg) and a 72-h oral administration (1330 mg/kg) and showed that in patients with early treatment (<10 h), the frequencies of hepatotoxicity were lower, such as 10%, 1.6%, and 6.1%, compared with patients treated later (>10–24 h), in which the risk of hepatotoxicity occurred with a higher frequency, such as 27.1%, 52.6%, and 26.4%. The authors concluded that all three regimens were equally effective when started within 10 h of overdose, even if the 48-h I.V. and 72-h oral regimens were superior in the patients treated later (Smilkstein et al., 1988; Smilkstein et al., 1991). The regimens and routes of administration of all studies are reported in Tables 1–3.

### N-Acetylcysteine Efficacy in Preventing Mortality

The 34 studies that reported the use of NAC in APAP-related DILI cases included a total of 19,580 patients. The mortality rate across different studies ranged from 0 to 52%. Among included patients, 2,376 developed hepatotoxicity.

Retrospective studies included 14,011 patients, of whom 320 died or underwent liver transplantation (data from studies ranged from 0 to 136), and 1,127 developed hepatotoxicities (data from studies ranged from 0 to 599). AEs were reported only in nine studies (Table 1).

Prospective studies included 5,299 patients, of whom 164 died or needed liver transplantation (data from studies ranged from 0 to 79), and 1,217 had hepatotoxicities (data from studies ranged from 0 to 611). AEs were reported in almost all studies, involving 385 patients (data from studies ranged from 0 to 147) (Table 2).

RCTs included 270 patients, of whom 13 died or underwent liver transplantation and 32 developed hepatotoxicities. AEs were reported in 47 patients (data from studies ranged from 0 to 36) (Table 3).

Outcomes were described as DILI mortality and/or liver transplantation and hepatotoxicity. Liver transplantation was reported in few studies (Harrison et al., 1990; Keays et al., 1991), which included patients who presented with ALF. In the retrospective study of Harrison et al. (1990), fulminant hepatitis showed a good outcome after late administration of NAC with a low mortality rate. In the trial conducted by Keays et al. (1991), NAC was found to be a safe and effective treatment for fulminant hepatic failure after APAP overdose, as mortality in the treatment group was 52%, while in the control group, mortality rose to 80%. In two RCTs, the co-administration of NAC with other drugs was investigated. In the RCT by Bateman et al. (2014), the schedule of NAC infusion (shorter vs. longer duration) with or without ondansetron to prevent AEs was compared in patients assigned randomly to different treatment schedules or placebo. In the patients randomized to a shorter NAC infusion duration and ondansetron administration, a higher incidence of hepatotoxicity was documented, whereas a lower incidence of AEs (especially nausea) was reported. In the RCT by Morrison et al. (2019), NAC was administered with calmangafodipir, a new chemotherapeutic agent, which was well tolerated, resulting in a rapid reduction of increased serum aminotransferases.

### N-Acetylcysteine Efficacy in Preventing Hepatotoxicity

As shown in Tables 1–3, hepatotoxicity by APAP overdose was reported in almost all studies (18 retrospective studies, 13 prospective studies, and 3 RCTs). Overall, 2,376 patients developed hepatotoxicity, ranging from 0 to 89% of the included patients in retrospective studies, from 0 to 53% in patients in prospective studies, and from 0 to 33% in patients enrolled in RCTs. However, among these studies, the pattern of liver injury was not ascertained due to the lack of data.

In the multicenter study by Smilkstein et al. (1988), which was the continuation of a previous study by Rumack et al. (1981), 2,540 patients underwent oral treatment with NAC and were classified based on the timing of treatment from APAP overdose. Nearly 26% of the patients who were treated within 10–24 h after APAP exposure developed hepatotoxicity, compared with the 6.1% who were treated within 10 h. Interestingly, in the study by Doyon and Klein-Schwarrz (2009), only 5.2% of patients developed hepatotoxicity following an overdose due to the early (within 8 h) treatment with an I.V. schedule of 21 h. It is noteworthy that in this study, stratification was quite different and the sample size was smaller compared with Smilkstein’s study.

Hepatotoxicity ranged from 32 to 37% in studies conducted in the obese population (Varney et al., 2014; Radosevich et al., 2016). It is noteworthy that in obese patients with a weight-based protocol, a larger dosage of NAC was administered compared with the standard regimens, without relevant AEs.

In the study by Alhelail et al. (2011), it has been reported that in patients with supratherapeutic ingestion of APAP, hepatotoxicity and death were related to the presence of alcohol abuse and high creatinine plasma levels. Schmidt et al. showed that chronic alcohol abuse was an independent risk factor for developing DILI and mortality after APAP overdose (Schmidt et al., 2002). Further, Myers et al. reported that long-term mortality was associated with preventable conditions, such as alcohol abuse and underlying liver disease (Myers et al., 2008). Thus, the development of hepatotoxicity by APAP was dependent not only on the acetaminophen dose ingested but also on the timing of treatment and additional risk factors, such as chronic alcohol abuse or underlying liver disease.

### N-Acetylcysteine Safety

AEs related to NAC administration were described in 18 studies (Bateman et al., 2014; Heard et al., 2014; Prescott et al., 1979; Harrison et al., 1990; Yip et al., 1998; Whyte et al., 2007; Yarema et al., 2009; Varney et al., 2014; Radosevich et al., 2016; Yarema et al., 2018; Buckley et al., 1999; Perry and Shannon, 1998; Smilkstein et al., 1991; Betten et al., 2007; Waring et al., 2008; Gheshlaghi, 2006; Keays et al., 1991; Morrison et al., 2019). AEs were mainly cutaneous and not severe, such as erythema, urticaria, itching, and anaphylactic reactions. Other AEs were abdominal pain, nausea, vomiting, dyspepsia, and fever, which might be caused by the APAP overdose itself; therefore, it is difficult to establish causality. Most of the studies reporting AEs used I.V. regimens, and anaphylactic reactions were mainly observed during the first hour of NAC infusion, corresponding to the peak of NAC concentrations, rather than errors in the preparation of the solutions. Allergic reactions, such as rash, pruritus, angioedema, or bronchospasm, have been reported, often associated with tachycardia or hypotension. These adverse effects were managed with steroids, antihistamines, and bronchodilators (Kerr et al., 2005).

Common risk factors identified for adverse reactions were female sex and a family history of allergy and asthma (Yarema et al., 2018). In the prospective study by Bateman et al., which aimed to decrease adverse effects, they attempted to reduce infusion times from 20 to 12 h after premedication of undergoing patients with ondansetron, an antiemetic drug. Reduction of administration time decreased both vomiting and anaphylactic reactions and made it easier to administer, since many of the AEs often occurred due to an erroneous preparation of the drug infusion. In patients with nausea and vomiting secondary to APAP overdose, the oral route was avoided, since it was impossible to administer the drug using this route. The reported rate of AEs among studies varied between 6 and 14.5% for anaphylactic reactions, whereas it ranged from 4 to 28% for less serious reactions, such as nausea and vomiting.

In the study by Varney et al. (2014), a weight-based NAC dose rather than a maximum weight cut-off dose was used. AEs were relatively common but not serious. In addition, Radosevich et al. showed that obese and nonobese patients treated with I.V. NAC for APAP overdose experienced similar rates of hepatotoxicity, 27.5 vs. 37.5% (Radosevich et al., 2016), respectively. AEs from each study are shown in Tables 1–3.

Hepatotoxicity as an AE to NAC administration has been reported only in the RCT by Bateman et al. (2014), in which a peak of ALT after NAC infusion was recorded in 22 patients (13 allocated to the standard NAC regimen and 9 to the shorter modified schedule). An escalation in the activity of alanine aminotransferase was more frequent in patients pretreated with ondansetron to prevent AEs (16 out of 100) compared with those pretreated with placebo. However, Bateman’s study was not powered to detect noninferiority of the shorter protocol versus the standard protocol, and further research is therefore needed.

### N-Acetylcysteine in Pregnant Women

Pregnant women with APAP overdose have been described in the study by Rigges et al. (1989), in which 60 patients were enrolled. In this study, 19 women overdosed during the first trimester, 22 during the second, and 19 during the third. Of 24 patients with APAP levels above the nomogram line, 10 were treated with the oral NAC protocol within 10 h after ingestion (8 normal delivery and 2 elective abortion), 10 were treated within 10–16 h after ingestion (5 delivered viable infants and 3 had elective and 2 spontaneous abortions), and 4 were treated after 16–24 h after ingestion (1 mother died, and there was a spontaneous abortion, 1 stillbirth, 1 elective abortion, and 1 delivery). By analysis, it has been shown that there was a statistically significant correlation between the time to loading of the NAC dose and pregnancy outcomes, with an increase in spontaneous abortion or fetal death when treatment began late. Seven years later, Horowitz et al. (1997) confirmed the abovementioned results, supporting the fact that pregnant women with APAP overdose should be treated with NAC as early as possible.

### N-Acetylcysteine in the Pediatric Population

APAP is commonly used to reduce fever in pediatric populations. The current dosage recommendations for APAP in children still largely rely on body weight. However, pediatric patients are not simply small adults, as they often require very different treatment because of their dynamic physiology, affecting pharmacokinetics.

Hepatotoxicity is the greatest concern associated with APAP treatment. A study carried out on 662 younger pediatric patients showed that they had less hepatotoxicity when exposed to the same levels of APAP acutely as older children and adults (Rumack et al., 1981). However, the pediatric ALF study group found that with chronic exposure to APAP (in contrast to a single toxic dose), younger patients (median age, 3.5 years) experienced a greater level of hepatotoxicity than older adolescent patients (median age, 15.2 years), who were more likely to experience hepatotoxicity from a single toxic dose (Rumack, 1984).

Among prospective cohort studies, the study by Perry and Shannon (Perry and Shannon, 1998), which included 54 adolescent patients, showed that there was no significant difference between treatment groups in age, sex, or distribution of risk categories. Hepatotoxicity was 8% in the I.V. NAC group and 7% in the oral NAC group. All patients recovered; thus, NAC administration in adolescents is safe, either as oral or I.V. administration.

## Discussion

This comprehensive review of clinical trials, prospective and retrospective cohort studies, and case series including 19,580 patients suffering from APAP overdose and APAP-related DILI shows that NAC prevents mortality and liver injury among treated patients. In addition, a large variability in the NAC schedule was found in both I.V. (ranging from 100 to 150 mg/kg) and oral regimens (ranging from 70 to 140 mg/pro Kg) and length of treatment (12 to 24–48 h for I.V. regimen; 21–72 h for oral regimen). The benefit of NAC was clear and clinically relevant, supporting the decision to treat all patients, independently of the evidence of liver injury (increases of aminotransferases). Only approximately 2,376 of 19,580 patients developed hepatotoxicity, thereby confirming the high efficacy of NAC treatment, even in pregnant women and children. Overall, safety in all studies with available information was acceptable, and patients did not show severe adverse reactions. Delaying treatment by 8–16 h and no more than 24 h from APAP overdose could reduce the efficacy of treatment, in terms of hepatotoxicity development, but did not affect mortality or the need for transplantation.

In our review, 2,376 patients developed hepatotoxicity, defined as an increase in aminotransferases of more than 1,000 IU/L (Heard et al., 2014). Most of the included studies did not consider pre-existing liver disease as a predisposing condition to worsen liver injury due to APAP overdose; thus, hepatotoxicity in some studies (Khandelwal et al., 2011; Betten et al., 2007) could be affected by not having recognized the presence of an underlying liver disease. Further, in obese patients, in whom a larger dosage of NAC should be used (Varney et al., 2014), steatosis and oxidative stress could have contributed to the development of liver injury. In addition, chronic alcohol abuse could be considered a risk factor for liver injury and death in patients with supratherapeutic APAP ingestion (Rumack and Matthew, 1975; Myers et al., 2008; Alhelail et al., 2011). Consumption of ethanol was reported in many other studies, even in those involving children under the age of 5 years (Rumack, 1984); in fact, at the time of that publication, many pediatric preparations of APAP contained ethanol. Thus, the rate of hepatotoxicity was high (50%) in pregnant women, in whom the late administration of NAC, caused by the unknown outcome during pregnancy, was probably responsible for the development of liver damage (Rigges et al., 1989; Horowitz et al., 1997).

The development of APAP-related DILI is mostly dependent on the dose of APAP ingestion and the timing of NAC administration. Although the dose is a good predictor of outcomes in these patients, experts currently recommend not exceeding 10 g or 200 mg/kg (Dart et al., 2006). At present, the gold standard for starting NAC treatment is a single-serum APAP concentration above the nomogram line of the Rumack-Matthew, between 4 and 24 h from ingestion (Prescott et al., 1975; Daly et al., 2008). However, patients with a history of supratherapeutic ingestion cannot be stratified with the Rumack-Matthew nomogram, and for malnourished patients or alcohol abusers, the line of serum APAP concentration is lower than that for treating patients without risk factors (Peterson and Rumack, 1978; Harrison et al., 1991; Rumack and Matthew, 1975; Bateman, 2015; Dufful and Isbister, 2013). The timing of NAC administration was reported in the majority of studies, either prospective or retrospective. All studies showed that early administration (<8–10 h) from APAP exposure is better than late administration (16–24 h) in terms of liver damage development and death. Further confirmation came from an Australian study that showed that NAC administration after an interval time ranging from 8 to 16 h after APAP ingestion determines an overall hepatotoxicity of 1.5% (23 out of 1571), being 0.4% in early admitted vs. 5.5% in late admitted patients (Kerr et al., 2005). Therefore, it is better to administer NAC, either oral or I.V., within 8 h from the APAP overdose.

Regarding the early administration of NAC and its antioxidant effect, it has been recently used to prevent non-APAP-related acute liver injury (due to viral, autoimmune, or indeterminate causes or drugs). In the study by Lee (Lee et al., 2009) published in 2009, it was shown that I.V. NAC improves transplant-free survival in the early stages of non-APAP-related ALF. In another study from Pakistan, carried out in a clinical setting in which liver transplant was not available and the main causes of ALF were viral infections, Mumtaz et al. showed that the administration of NAC was safe, and it was associated with a reduction in mortality (Mumtaz et al., 2009). In pediatric patients with non-APAP-related ALF, results are controversial even if NAC administration was associated with a shorter length of hospital stay, higher incidence of native liver recovery, and better survival after LT (Squires et al., 2013). However, due to its ability to improve hemodynamics and oxygenation of liver tissue via varying antioxidant mechanisms, NAC was safe even for non-APAP-related ALF, since it prolongs survival with a native liver and after liver transplantation. Nonetheless, it should be noted that NAC for non-APAP, although it may improve overall survival and lower the risk of transplantation in some subgroups of patients, is not widely validated and recommended for clinical practice (Chughlay et al., 2016).

Another still debated issue is regarding the route of the NAC regimen, oral or I.V.; in fact, there are no RCTs comparing the two modes of administration. In our review, we found that six cohort studies reported a comparison between the routes of NAC administration (I.V, and oral). In the study by Yip et al. (1998), 76 patients were administered intravenously with an oral preparation. Administration of an oral NAC preparation intravenously appears to have limited AEs and offers another mechanism of delivery of the potentially lifesaving NAC when oral administration is not possible. It seems that oral administration, rarely used in the United States and Europe, is a matter of dose intensity (Yarema et al., 2009) rather than route, as reported by Rumack and Bateman in 2012 (Rumack and Bateman, 2012). The oral protocol resulted in a dose of 17.5 mg/kg/h delivered to the liver following a loading dose for a total of 72 h. Oral NAC is absorbed directly into the splanchnic flow and produces higher levels in the liver, but due to the first pass, lower levels in the blood than I.V. The I.V. administration of NAC does not deliver the same amount to the liver, although it provides a blood level that is higher than that provided by oral administration. In fact, the total dose of NAC administration in the oral protocol (1,330 mg/kg over 72 h) is higher than that in the I.V. protocol (300 mg/kg over 20 h).

Oral NAC administration is common for patients with preclinical damage, who reported accidental supratherapeutic APAP ingestion, and for those with levels of APAP higher than the nomogram line; treatment should be stopped when ALT levels decrease and serum APAP concentrations are undetectable. By contrast, patients arriving unconscious to the emergency room, after 8 h from the APAP overdose, needing to be admitted to the critical care unit with frequent monitoring of vital signs and with ongoing liver damage, should be treated with the I.V. schedule (Bateman et al., 2014).

Whether there are differences in the treatment of acute paracetamol overdose versus unintentional intoxication at therapeutic doses over any period is not well known. Patients with unintentional acute intoxication had a worse outcome in relation to the development of ALF and a greater number of hospitalization days (Piotrowska et al., 2019; Rotundo and Pyrsopoulos, 2020). This is probably because these patients are hospitalized late, as many of them take APAP unknowingly (analgesic compounds, opioids, antihistamines), often exceeding the permitted threshold doses.

However, the difference between intentional and unintentional acute intoxication is determined by a psychiatrist and cannot be determined by laboratory levels or clinical toxicologists. Experienced centers are informed that it is important to cure the patient and not the intoxication, by matching the use of NAC and supportive therapies. A balance between the safety of NAC dosing in the majority of low-risk patients must be considered at the same time as an increased dose intensity and duration in potentially more severely poisoned patients. Rumack and Bateman (2012) reported that in significant cases (ALT change 24 h after the overdose and increased INR), a prolonged duration of therapy over 21 h, delivering a higher dose of NAC, monitored by appropriate clinical end-points to avoid AEs, will result in the best possible outcome for the patients.

In recent times, to overcome the difficulties due to the poor bioavailability of NAC, new molecules such as fomepizole (Kang et al., 2020) and N-acetylcysteine-amide (NACA) have been studied (Khayyat et al., 2016). The first molecule, fomepizole, a cytochrome P450 inhibitor, has been tested in a crossover trial in which six adults were successfully treated with 4-methylpyrazole (I.V. 15 mg/kg, followed by 10 mg/kg 12 h later) after ingestion of a supratherapeutic APAP dose to prevent hepatotoxicity. NACA is a novel antioxidant, that with a lower dose than NAC can prevent APAP-induced hepatotoxicity in mice by increasing glutathione levels. It would be very important to develop and test such drugs, because in the near future, they could represent a more effective and safer option for the prevention of APAP-induced toxicity; however, more research is needed.

Thus, in our study, we also included pregnant women and pediatric populations. Rigges et al. (1989), describing pregnant women with APAP overdose, showed that there was a statistically significant correlation between the time to loading of the NAC dose and pregnancy outcomes, with an increase in spontaneous abortion or fetal death when treatment began late. These results were confirmed afterward by Horowitz et al. (1997). Therefore, pregnant women with APAP overdose should be treated with NAC as early as possible.

The limitations of the current study are mainly due to the large heterogeneity and poor quality assessment of the analyzed studies.

In conclusion, treatment with NAC, if given within 8 h from APAP ingestion and no later than 24 h, may prevent or minimize liver damage. Oral and I.V. administration regimens are efficacious, even if they deliver a different amount of dose; thus, choosing the best route depends on the clinical severity of the patient. At present, because NAC treatment represents a real and effective preventive measure for any liver damage and ALF requiring liver transplantation, good clinical practice in any emergency department, clinic, or hospital still requires a high degree of medical suspicion and laboratory testing of the APAP overdose.

## Data Availability

The original contributions presented in the study are included in the article/supplementary material; further inquiries can be directed to the corresponding author.

## Abbreviations

AEs, adverse events; ALF, acute liver failure; APAP, acetaminophen; ALT, alanine aminotransferase; AST, aspartate aminotransferase; DILI, drug-induced liver injury; GGT, gamma-glutamyl transferase; NACA, N-acetylcysteine-amide; NAC, N-acetylcysteine; RCTs, randomized clinical trials.
